# Personalized 3D-Printed Bioresorbable Airway External Splint for Tracheomalacia Combined With Congenital Heart Disease

**DOI:** 10.3389/fbioe.2022.859777

**Published:** 2022-05-10

**Authors:** Di Yu, Wei Peng, Xuming Mo, Yuxi Zhang, Xing Zhang, Jiankang He

**Affiliations:** ^1^ Department of Cardiothoracic Surgery, Children’s Hospital of Nanjing Medical University, Nanjing, China; ^2^ State Key Laboratory for Manufacturing Systems Engineering, Xi’an Jiaotong University, Xi’an, China

**Keywords:** tracheomalacia, splint, three-dimensional printing, congenital heart, follow-up

## Abstract

Severe tracheomalacia (TM) patients with respiratory symptoms need surgical intervention, including aortopexy, internal stents or external splint. While some patients continue to have respiratory symptoms after tracheal relief, and there is no evidence to support any one surgery therapy over another. Here we introduce a clinical safety and efficacy of the three-dimensional (3D)-printed bioresorbable airway external splints in treating congenital heart disease (CHD) patients with severe TM. From May 2019 to September 2020, nine patients with severe TM were enrolled. The median age was 5 months (range, 3–25 months), and the median weight was 7.5 kg (range, 3–15 kg). All patients had wheezing, and two patients were assisted by machine ventilation (MV) preoperatively. The median length of TM was 1.5 cm (range, 1.0–3.0 cm). All patients underwent suspension of a “C”-shaped lumen airway external splint, which were designed in SOLIDWORKS and made of polycaprolactone (PCL). The airway external splint could provided effective support for at least 6 months and was completely degraded into carbon dioxide and water within 2–3 years. The median time of postoperative machine assisted ventilation was 23.7 h (range, 3.3–223.4 h), and the median time of ICU stay was 9 days (range, 4–25 days). The median follow-up time was 18 months (range, 12–24 months). Respiratory symptoms were all relieved, and no external splint-associated complications occurred. The 3D computed tomography reconstruction showed no airway stenosis. Personalized 3D-printed bioresorbable airway external splint can not only limit external compression and prevent airway collapse but also ensure the growth potential of the airway, which is a safe, reliable and effective treatment for CHD with TM.

## Introduction

Tracheomalacia (TM) refers to an excessive laxity of the pars membranacea or deficiency in the integrity of cartilage, leading to the collapse and narrowing of the tracheal lumen >50% during expiration (Lee et al., 2008). Tracheobronchomalacia (TBM) is a condition in which the main bronchi are also affected, while only one or both of the main bronchi are affected in bronchomalacia (BM) (Carden et al., 2005). TM has an estimated incidence of approximately 1:2000 and is commonly divided into congenital and acquired TM (Hysinger et al., 2020). Congenital or primary TM is usually associated with a syndrome, while acquired or secondary TM is related to extrinsic tracheal compression, especially for patients with congenital heart disease (CHD), such as vascular ring and enlarged heart (Carden et al., 2005).

Flexible bronchoscopy is recommended for use as the “gold standard” diagnostic test by the European Respiratory Society (ERS), and the degree of TM can be arbitrarily described as mild (50–75% reduction), moderate (75–90% reduction) or severe (>90% reduction) (Wallis et al., 2019). Three-dimensional computed tomography (3D-CT) reconstruction provides excellent anatomic details of the tracheal and adjacent structures, although the children are exposed to ionizing radiation. Nevertheless, it is a better choice for TM patients with CHD, as it can fully assess the vascular structure and inner cardiac malformations.

Observation and conservative management are the preferred therapies for TM, while surgical intervention is necessary for severe TM and all the associated conditions, such as vascular anomalies and affected regions of the trachea, should be considered (Fraga et al., 2016; Kamran and Jennings, 2019). Surgical options for the treatment of TM include aortopexy, tracheal resection, internal stents and external splints. However, aortopexy has shown no improvement or even worsening (Torre et al., 2012), and the formation of granulation tissue was found after endotracheal stent implantation in some patients (Furman et al., 1999). Moreover, some children with TM caused by vascular compression continue to have severe respiratory symptoms after vascular relief and require another surgical treatment (Fraga et al., 2009). Therefore, this study introduces a new surgical option, the personalized 3D-printed bioresorbable airway external splint, and evaluates its feasibility and effectiveness for TM combined with CHD.

## Subjects and Methods

### Subjects

This airway external splint is not for commercial use but was approved by the Children’s Hospital of Nanjing Medical University Human Research Ethics Committees. All patients underwent flexible bronchoscopy to confirm TM/TBM/BM, and patients with anatomic stenosis including complete tracheal ring were excluded. A multidisciplinary team composed of cardiac surgeons, respiratory physicians, intensive care physicians, and radiologists, was established to evaluate the degree of airway malacia preoperatively. The patients were enrolled if they still needed continuous positive airway pressure (CPAP) or ventilator assistance for a long time postoperatively after evaluation. They were told about the airway external splint and provided written consent. A total of nine patients with TM/TBM/BM related to extrinsic tracheal compression caused by CHD were included from May 2019 to September 2020.

### Design of Splint

CT of the patient’s airway was performed using a 64-slice multidetector-row CT scanner (Brilliance iCT, Philips, Netherland). All axial CT images were obtained with a 0.5-mm slice thickness. These image files were saved in Digital Imaging and Communications in Medicine (DICOM) format and then imported into Mimics software (Materialise Co., Belgium) for airway model reconstruction. Patient-specific external airway splints with a “C”-shaped lumen were then designed in SOLIDWORKS (Dassault Systèmes Corp. France). The splint diameter, length, opening angle and pore were customized according to the reconstructed malacic airways. Afterward, the designed splints were converted in. STL format and loaded into a selective laser sintering system (SLS, Xi’an Jiaotong University, China) for manufacturing, which is equipped with a laser with a spot diameter of 60 μm. Polycaprolactone (PCL) with a molecular weight of 80,000 was purchased from Jinan Daigang Biomaterial Co., Ltd. (China). PCL with an average size of 100 μm was employed for the SLS process due to its excellent biocompatibility, biodegradability and relatively robust mechanical properties, which can maintain support for the malacic airway for a long time. The structural design of the splints was similar with previous studies, which was projected as thread type with a thickness of 1 mm and an opening ring of 240° (Huang et al., 2016; Wang et al., 2019). Before surgical implantation, all splints were sterilized with cobalt-60.

### Procedure

Two patients with a right aortic arch (RAA) with aberrant left subclavian artery (ALSCA) underwent surgery through the left fourth intercostal space, and the ligamentum arteriosum was fully freed and divided without cardiopulmonary bypass (CPB). The other seven patients underwent median sternotomy, and CPB was initiated in six patients. The left pulmonary artery was resected from the right pulmonary artery, transferred to the left of the trachea, and then implanted in the left side of the main pulmonary artery in pulmonary artery sling (PAS) patients. The left arch was divided in double aortic arch (DAA) patients. The inner cardiac defect was repaired simultaneously. Then, the trachea and/or bronchi were carefully and fully freed. The length and position of the malacia airway were determined by flexible bronchoscopy (FB-10 V, Pentax, Japan). For the selection of the size of the external splint, the length and inner diameter of the tracheal stenosis were measured through three-dimensional CT reconstruction of the trachea before the operation. When printing the external splint in 3D, the length was 5 mm longer than the narrow segment, and the inner diameter was 5 mm larger than the inner diameter of the normal trachea at the proximal end of the narrow segment. During the operation, the surgeon can further modify the splint according to the anatomy of the child. The airway external splint was fixed on the cartilage at the four corners through the pores, and the collapsed pars membranacea was then suspended on the splint with interrupted anastomosis. During the operation, flexible bronchoscopy was used to observe whether the sutures penetrated the cartilage and pars membranacea, and the airway stenosis was relieved. Two patients underwent aortopexy.

### Follow-Up

All patients were followed up by flexible bronchoscopy within 3 days after withdrawal of mechanical ventilation to evaluate the degree of airway malacia. After discharge, all patients were followed up at 1, 3, 6, 12, 18 and 24 months. Chest X-ray and echocardiography were performed, and respiratory symptoms were assessed. Chest 3D-CT reconstruction of the trachea and bronchi was performed 12 and 24 months after discharge to observe whether there was airway stenosis.

### Statistical Analysis

Data are expressed as the median [interquartile range (IQR)]. Statistical analysis was performed with IBM SPSS v22 (IBM Corp. Armonk, NY). The Wilcoxon test was performed to compare the degree of malacia before and after the operation. Statistical significance was set at *p* < 0.05.

## Results

### Patient Characteristics

The median age of the patients was 5 months (IQR, 3.5–14.5 months), and the median weight was 7.5 kg (IQR, 3.6–9.7 kg). All patients had respiratory symptoms and wheezing, and two of them had recurrent respiratory tract infections. Preoperative echocardiography showed that four of the patients were diagnosed with inner cardiac defects and 6 with vascular rings. Trachea and bronchi 3D-CT reconstruction and flexible bronchoscopy were performed in all patients, and all of them were diagnosed with severe tracheal and/or bronchial stenosis and severe TM/TBM/BM, respectively. Ultimately, 4, two and three patients were diagnosed with TM, TBM and BM, respectively. The notable malacia site of trachea, left bronchus and right bronchus were found in 6, 2 and 1, respectively. The median length of TM was 1.5 cm (IQR, 1.0–1.5 cm), ranging from 1.0 to 3.0 cm, and the median narrowest diameter of TM was 1.2 mm (IQR, 0.9–2.1 mm). Two patients were assisted by machine ventilation (MV) preoperatively. The baseline characteristics of the patients are shown in Table 1.

**TABLE 1:** Characteristic of patients with 3D-printed bioresorable airway external splint.

Patient	Age (month)	Weight (kg)	Gender	Diagnosis	TM/TBM/BM	Preoperative MV support	Malacia site	Malacia long (cm)	Degree of malacia	Narrowest diameter of malacia airway (mm)
1	3	7.5	Female	PAS	TBM	No	Trachea	3.0	Severe	1.1
2	4	3.5	Male	TOF	TM	Yes	Trachea	1.0	Severe	1.2
3	25	15	Male	PAS	BM	No	Left Bronchus	1.5	Severe	1.4
4	3	3	Female	VSD/ASD/BPD	TM	Yes	Trachea	1.5	Severe	1.2
5	13	9.5	Male	RAA/ALSCA	TM	No	Trachea	1.5	Severe	1.4
6	5	6.3	Female	VSD/ASD/PDA	BM	No	Left Bronchus	1.5	Severe	0.9
7	4	3.7	Male	PAS	TBM	No	Trachea	1.0	Severe	1.4
8	12	10	Male	RAA/ALSCA	BM	No	Right Bronchus	1.0	Severe	1.1
9	16	9.5	Female	DAA/VSD/RVOTO/PS	TM	No	Trachea	1.0	Severe	2.1

TM, tracheomalacia; TBM, tracheobronchomalacia; BM, bronchomalacia; PAS, pulmonary artery sling; TOF, tetralogy of fallot; VSD, ventricular septal defect; ASD, atrial septal defect; BPD, bronchopulmonary dysplasia; RAA, right aortic arch; ALSCA, aberrant left subclavian artery; PDA, patent ductus arteriosus; DAA, double aortic arch; RVOTO, right ventricular outflow tract obstruction; PS, pulmonary stenosis.

### Clinical Outcomes

**FIGURE 1:**
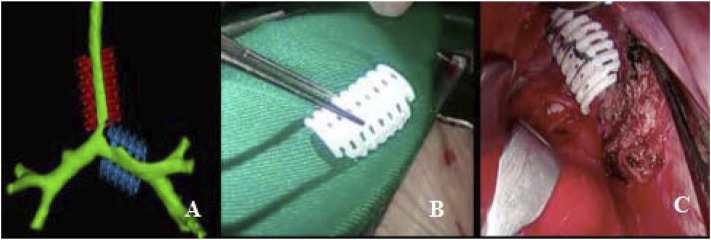
Personalized 3D-printed bioresorbable airway external splint. **(A)** Virtual assessment of fit of the splints over airway model. **(B)** Three-dimensional-printed bioresorbable airway external splint. **(C)** Intraoperative placement of the splint overlying the malacia tracheal segment.

One personalized 3D-printed bioresorbable airway external splint was used for each patient. Six patients were implanted in the trachea; 2, in the left bronchus; and 1, in the right bronchus. The median length of airway external splint was 2.0 cm (IQR, 1.5–2.0 cm), ranging from 1.5 to 3.5 cm. Four patients had inner cardiac defects repaired under CPB, and six patients underwent vascular ring division. One patient underwent only airway external splinting and aortopexy. No external splint-associated complications occurred. The median time of MV was 23.7 h (IQR, 3.9–61.2 h), and the median time of ICU stay was 9 days (IQR, 6.5–10.0 days). Postoperative flexible bronchoscopy showed no tracheal and/or bronchial stenosis or malacia (Figure 1), and the respiratory symptoms were relieved in all patients; only one patient showed slight wheezing. Postoperative 3D-CT reconstruction showed the median narrowest diameter of TM was 3.4 mm (IQR, 2.8–4.5 mm). The outcomes of operation are showed in Table 2.

**TABLE 2:** Outcomes of operation.

Patient	Cardiovascular operation	Aortopexy	CPB time (minute)	ACC time (minute)	MV time (hour)	ICU stay (day)	Malacia	Narrowest diameter of malacia airway (mm) after operation	Complications	Follow-up time (month)
1	Left pulmonary artery re-implantation	No	130	0	73.2	10	No	3.2	No	24
2	TOF repair	No	90	48	42.2	9	No	3.4	No	24
3	Left pulmonary artery re-implantation	No	100	0	3.9	6	No	3.0	No	24
4	VSD repair + ASD repair	Yes	80	35	23.7	9	No	3.3	No	24
5	Vascular ring division	Yes	0	0	5	4	No	3.5	No	18
6	VSD repair + ASD repair + PDA ligation	No	90	40.5	223.4	25	No	2.8	No	18
7	Left pulmonary artery re-implantation	No	0	0	49.3	10	No	3.4	No	12
8	Vascular ring division	No	0	0	3.3	7	No	3.6	No	12
9	Left aortic arch division + VSD repair + reconstruction of RVOT	No	98	36.7	3.9	10	No	4.5	No	12

CPB, cardiopulmonary bypass; ACC, aortic cross-clamping; MV, machine ventilation; ICU, intense care unit; TOF, tetralogy of fallot; VSD, ventricular septal defect; ASD, atrial septal defect; PDA, patent ductus arteriosus; RVOT, right ventricular outflow tract.

### Complications

No external splint-associated complications, including foreign body reaction, infection, and erosion into nearby structures (Sood et al., 2021), were found in any patients.

### Follow-Up

**FIGURE 2:**
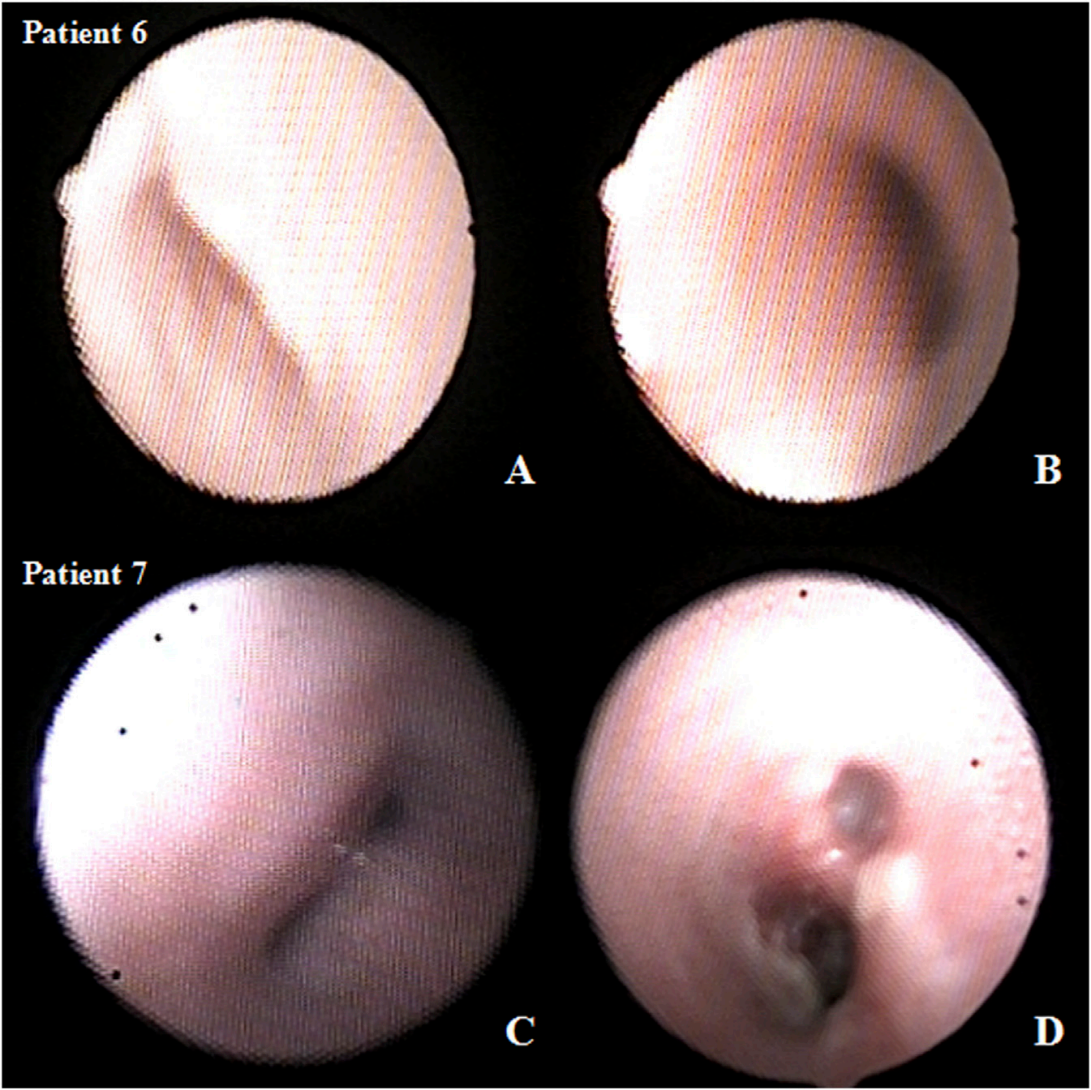
Preoperative and postoperative three-dimensional computed tomography (3D-CT) images after airway external splint suspension. **(A)** preoperative and **(B)** 1-year after operation 3D-CT images in patient six and **(C)** preoperative and **(D)** 1-year after operation 3D-CT images in patient 7.

**FIGURE 3:**
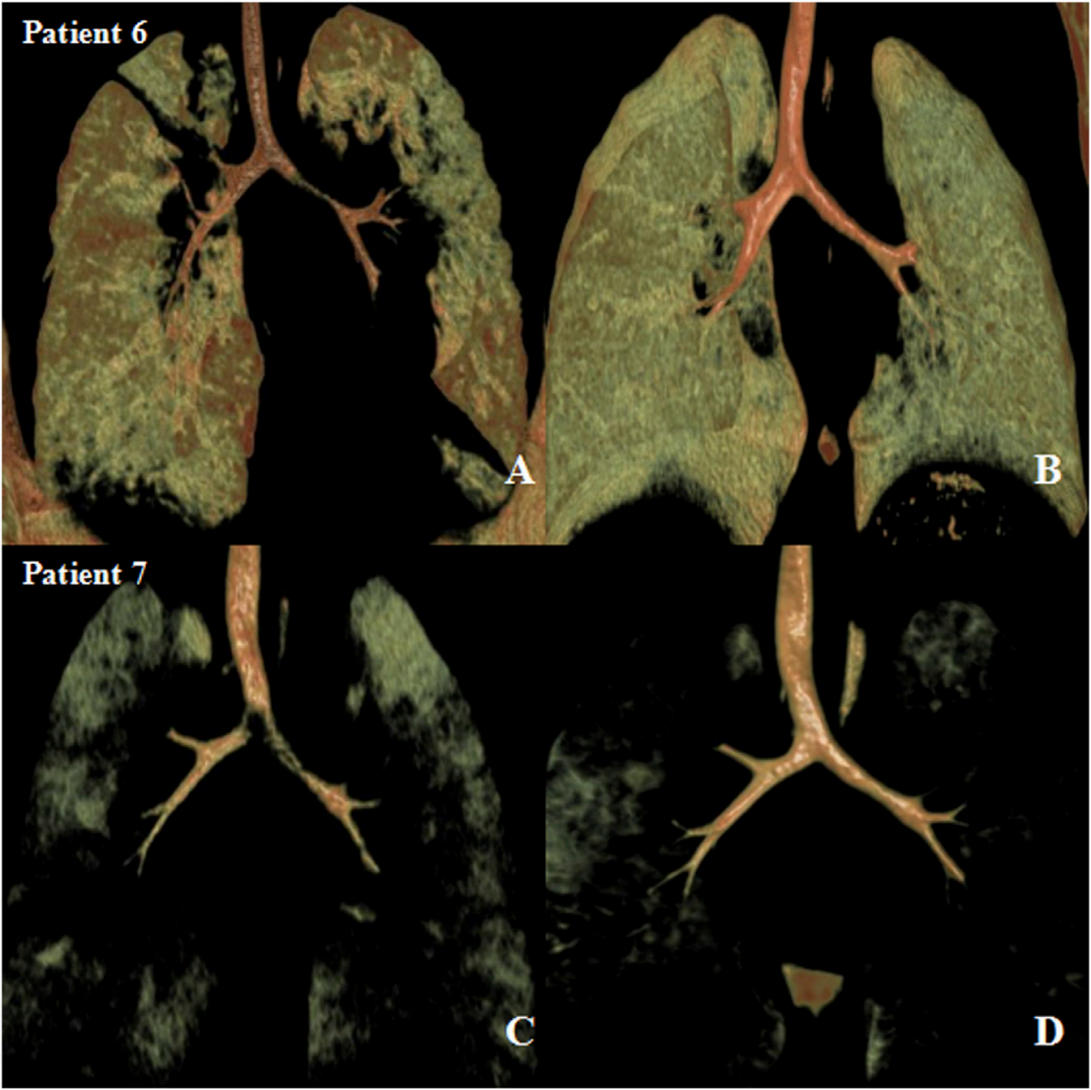
Preoperative and postoperative bronchoscopy after airway external splint suspension in the patient. **(A)** preoperative and **(B)** postoperative bronchoscopy in patient six and **(C)** preoperative and **(D)** postoperative bronchoscopy in patient 7.

The median follow-up time was 18 months (IQR, 12–24 months). Eight patients had resolution of respiratory symptoms, and one patient still had slight wheezing during the 12-months follow-up. The tracheal and bronchial 3D-CT reconstruction showed no tracheal and/or bronchial compression or stenosis (Figures 2, 3).

## Discussion

CHD in children can be complicated with acquired tracheomalacia, which is mainly caused by specific vascular anatomical malformations, such as DAA, PAS, and RAA with ALSCA, or compression caused by an enlarged heart (Fraga et al., 2016). For most patients, after compression of the vasculature and/or heart was released, tracheomalacia was gradually ameliorated, and respiratory symptoms improved. However, without surgery, patients with severe tracheomalacia or TM combined with bronchopulmonary dysplasia would need to rely on lifelong CPAP or MV.

The most common surgery is aortopexy. However, a meta-analysis of aortopexy reported that more than 80% of patients showed significantly improved symptoms after aortopexy, while 8% showed no improvement, 4% had a worsening of symptoms and 6% died. Moreover, the overall complication rate was 15%, and 1% needed redo aortopexy (Torre et al., 2012).

Endoscopic placement of internal tracheal stents has been attempted to treat TM/TBM in children with the advantages of less invasiveness and shorter recovery. Due to the growth of the children, the stents were difficult to remove, or larger stents were needed (Fraga et al., 2016). Moreover, complications, such as granulation tissue formation, erosion into nearby structures and migration, have also been reported (Antón-Pacheco et al., 2008; Wallis and McLaren, 2018). Valerie reported that airway stents resulted in a higher incidence of cardiac arrest and even death than aortopexy (Valerie et al., 2005). In China, several researchers have tried to use coronary stents, renal artery stents and biliary stents as internal tracheal stents and have achieved good outcomes; however, migration and granulation tissue formation cannot be avoided (Yang et al., 2019). Therefore, airway stents are used in very limited situations for TM/TBM in children.

In some patients with severe long segmental TM/TBM and aortopexy, the symptoms may not be alleviated, and external splinting may be applied to support the malacic trachea with autologous and prosthetic materials. However, complications including infection and erosion into nearby structures were also considered (Shieh and Jennings, 2017). Other intervention therapies, such as tracheobronchopexy (Bairdain et al., 2016), end-to-end tracheal resection and slide tracheoplasty, are not widely used (Kamran and Jennings, 2019). However, according to the Cochrane review of the interventions for primary TM in children in 2012, there is no evidence to support any one therapy over another for the treatment of TM (Goyal et al., 2012).

In 2013, Zopf et al. (2013) first reported a critically ill patient with severe TBM who was treated with a 3D printed bioresorbable airway splint made of PCL, with good anticollapse, bending, extension and expansion abilities. This splint was expected to be absorbed within 2–3 years and obviate the need for surgical removal. Dr Green’s group from the University of Michigan used this splint for 15 critically ill children with severe TBM, and the children experienced a good clinical benefit with a lower rate of mortality and complications (Morrison et al., 2015; Les et al., 2019). In addition, Liu et al. (2020) used a U-shaped microplate made of another absorbable material, poly-l-lactic acid (PLLA), to treat patients with severe BM and showed a good outcome. Absorbable airway splints seem to be a safe and effective treatment for TM/TBM.

For patients with TM/TBM compressed by an enlarged heart or vascular ring, the malacia could not immediately be alleviated after relieving the compression, and recovery would take several months. Airway stents and external splints can effectively solve this problem but need reoperation to remove it; otherwise, they would affect growth and development. The airway external splint prepared by 3D printing technology is customized according to the shape of each child’s trachea. The biodegradable material PCL is used, which can not only meet the performance requirements of extratracheal stents, such as shape, toughness, strength and elasticity, but also meet the specific requirements of human biocompatibility and degradability. After the external splint is implanted into the patient, it can provide effective support for at least 6 months and undergoes complete degradation into carbon dioxide and water within 2–3 years (Low et al., 2009; Meng et al., 2020). Therefore, bioresorbable airway external splints can not only limit external compression and prevent airway collapse but also ensure the growth potential of the airway.

In conclusion, we introduced a personalized 3D-printed bioresorbable airway external splint, which is a safe, reliable and effective treatment for CHD with TM/TBM; this may become an attractive treatment option in the future.

## Data Availability

The raw data supporting the conclusion of this article will be made available by the authors, without undue reservation.
